# A bacteriophage cocktail can efficiently reduce five important *Salmonella* serotypes both on chicken skin and stainless steel

**DOI:** 10.3389/fmicb.2024.1354696

**Published:** 2024-03-04

**Authors:** Tamar Gvaladze, Hansjörg Lehnherr, Stefan Hertwig

**Affiliations:** ^1^Department of Biological Safety, German Federal Institute for Risk Assessment, Berlin, Germany; ^2^Phage Technology Center GmbH, Bönen, Germany

**Keywords:** *Salmonella*, phage, biocontrol, chicken, steel, post-harvest application

## Abstract

*Salmonella* is one of the most important zoonotic pathogens and is mostly transmitted through food of animal origin. Application of bacteriophages is a promising tool to biocontrol *Salmonella* on both food and food contact surfaces. In this study, we evaluated the effectiveness of a six-phage cocktail for the reduction of *Salmonella* Enteritidis and a mixture of five major *Salmonella* serotypes (*S*. Enteritidis, *Salmonella* Typhimurium, *Salmonella* Infantis, *Salmonella* Paratyphi B, and *Salmonella* Indiana) on chicken skin and stainless steel. A phage cocktail with a final concentration of 10^7^ PFU/cm^2^ was sprayed on these surfaces. After adding the phage cocktail, the samples were incubated at RT (~23°C) for different periods of time. The phage cocktail caused a significant reduction of *S*. Enteritidis and the mixed culture on chicken skin 30 min after phage addition, with 1.8 log_10_ and 1 log_10_ units, respectively. Reduction rates (1.2–1.7 log_10_ units) on stainless steel after 30 min were similar. Four hours after addition, the phage cocktail caused a significant reduction on both surfaces up to 3 log_10_ units on chicken skin and 2.4 log_10_ units on stainless steel. In a further experiment, bacteria added to stainless steel were not allowed to dry to simulate a fresh bacterial contamination. In this case, the bacterial count of *S*. Enteritidis was reduced below the detection limit after 2 h. The results demonstrate that this phage cocktail has potential to be used in post-harvest applications to control *Salmonella* contaminations.

## 1 Introduction

*Salmonella* is a gram-negative, facultative anaerobic bacterium belonging to the *Enterobacteriaceae* family (Bahrani-Mougeot et al., 2009). It is classified into two species, *Salmonella enterica* and *Salmonella bongori*, and encompasses more than 2.600 serotypes (Issenhuth-Jeanjean et al., 2014). Serotypes of *Salmonella enterica* are of particular importance for human health as they are primarily associated with foodborne illnesses (Park and Andam, 2020). The illness caused by non-typhoidal *Salmonella* spp. (NTS) is called salmonellosis. The majority of outbreaks and sporadic cases of salmonellosis are attributed to contaminated food (Chanamé Pinedo et al., 2022; Teklemariam et al., 2023). Primary sources are poultry, raw and undercooked meat, egg and egg products and raw dairy products (Heredia and García, 2018).

In the European Union, 773 foodborne disease outbreaks occurred in 2021, with approximately 60,000 human cases (EFSA and ECDC (European Food Safety Authority and European Centre for Disease Prevention and Control), 2022). Up to 80% of illnesses are sporadic and not associated with outbreaks (Gut et al., 2018). The serotypes *Salmonella* Enteritidis and *Salmonella* Typhimurium are of significant importance, as they are mostly implicated in food-borne infections and outbreaks (Park and Andam, 2020). However, the prevalence of *Salmonella* in broiler farms is progressively linked to the *S*. Infantis serotype (Hauser et al., 2012; Mughini-Gras et al., 2021). After *S*. Enteritidis and *S*. Typhimurium, *Salmonella* Infantis is the third most frequently detected serotype in humans (Garcia-Soto et al., 2020). Furthermore, when compared to *S*. Enteritidis and *S*. Typhimurium, *S*. Infantis exhibited a significantly higher occurrence of persistent infections (Marzel et al., 2016). In addition, *Salmonella* Paratyphi B and *Salmonella* Indiana are epidemiologically important serotypes linked to chicken meat in Germany (Federal Office of Consumer Protection and Food Safety (BVL), 2020). Numerous positive cases of *S*. Paratyphi B and *S*. Indiana have been detected in poultry meat across various continents (Ferrari et al., 2019). Thus, *Salmonella* spp. are predominantly found in poultry products, with chicken being the primary reservoir (Ferrari et al., 2019; EFSA and ECDC (European Food Safety Authority and European Centre for Disease Prevention and Control), 2022). Although measures such as surveillance, biosecurity, and vaccination are adopted, salmonellosis remains a significant challenge for both public and animal health (Vandeplas et al., 2010; Ferrari et al., 2019). The safety of chicken meat products may be increased using chemical or physical antimicrobial measures (Wessels et al., 2021; Han et al., 2022). In this context, the use of antibiotics is of major concern, due to the global challenge of antibiotic resistance (Mehdi et al., 2018). Hence, it is of significant importance to enforce different control and preventive strategies to limit bacterial contamination, including *Salmonella*, in chicken-derived products.

The use of strictly lytic (virulent) bacteriophages, commonly designated as phages, represents an alternative approach, serving as a promising biocontrol tool to reduce agents either in living animals or during postharvest processing (Moye et al., 2018; Islam et al., 2021). Bacteriophages are viruses that infect exclusively bacteria by binding to specific cell wall receptors, followed by lysis of the bacterial cell. Phages are the most occurring biological entities in the biosphere (Clokie et al., 2011). There are temperate and virulent phages. Virulent phages undergo only a lytic cycle and are therefore utilized in therapy or for biocontrol (Sulakvelidze et al., 2001; Moye et al., 2018). Phages were discovered before antibiotics, but after the advent of antibiotics, phage research moved to the background (Clokie et al., 2011). Shortly after the discovery of antibiotics, the issue of antibiotic resistance emerged (Kutateladze and Adamia, 2010). Nowadays there is a global problem of antibiotic resistance leading to a revival of alternative approaches like harnessing phages (Willyard, 2017; Vikram et al., 2021). Phages, as antimicrobial agents, can be used for the therapy of both humans and animals, as well as for applications along the food chain (Hagens and Loessner, 2007; Abedon et al., 2014). Besides their specificity and the fact that they do not harm the normal microflora on food, one other advantage of using phages is that they do not change the organoleptic characteristics of food (Moye et al., 2018).

The use of phages in the poultry and chicken meat industry is currently receiving considerable attention. Several reviews have addressed this area of research in depth, particularly focusing on *Salmonella* spp. as target bacteria (Żbikowska et al., 2020; Islam et al., 2021; Wessels et al., 2021; Han et al., 2022; Khan and Rahman, 2022; Abd-El Wahab et al., 2023; Shahdadi et al., 2023). To date, there are a number of reports describing experimental studies on the reduction of *Salmonella* through post-harvest application (Goode et al., 2003; Hungaro et al., 2013; Duc et al., 2018; Atterbury et al., 2020; Esmael et al., 2021; Shakeri et al., 2021). In most studies, two *Salmonella* serotypes and a single phage or a phage cocktail were applied in a laboratory setting to chicken skin or to chicken meat. However, phage application is not confined to the research area; it is also effectively integrated into practical food processing. Among other commercially available phage products, the U.S. Food and Drug Administration (FDA) approved different *Salmonella* specific phage preparations for utilization within the poultry industry (Han et al., 2022). Several studies reported on the successful application of commercial *Salmonella* phages at the laboratory scale (Sukumaran et al., 2015, 2016; Grant et al., 2017; Yeh et al., 2017, 2018). In the USA and other developed countries, the use of phage cocktails with a Generally Recognized as Safe (GRAS) status is permitted. Conversely, within the European Union (EU), the application of phage preparations within the food industry is not allowed due to regulatory concerns (Abd-El Wahab et al., 2023). However, the use of phages to combat Listeria is regarded as safe within the EU (EFSA BIOHAZ Panel, 2016).

In this study, we evaluated the efficacy of a commercially available *Salmonella* phage product containing six phages on both chicken skin and stainless-steel surfaces. Some properties of the phages have already been described (Gvaladze et al., 2023). Four phages exhibited a myoviridal morphology, while the remaining two are siphoviruses. Five of them revealed a broad host range and a strong lytic activity on several *Salmonella* serotypes (*S*. Enteritidis, *S*. Typhimurium, *S*. Infantis, *S*. Paratyphi B, and *S*. Indiana) at low temperatures and at low MOIs in liquid cultures. Moreover, a mixture of ten strains belonging to five serotypes, was efficiently lysed down to 6°C. Here, the phages' ability to lyse these *Salmonella* serotypes on hard surfaces was studied. Stainless steel was selected as it is a common component of equipment used for food production. To our knowledge, only little data have been published yet on the application of phages on this surface. In addition, most studies used only *S*. Enteritidis and *S*. Typhimurium as the target serotypes.

## 2 Material and methods

### 2.1 Bacterial strains and growth conditions

The *Salmonella* strains used in this study were isolated from chicken products and stored at the National Reference Laboratory for *Salmonella*, German Federal Institute for Risk Assessment (BfR) Berlin, Germany (Table 1). *Salmonella* stock cultures were preserved at −80°C using glycerol as a cryoprotectant. Bacteria were grown overnight on lysogeny broth agar (LB; Carl Roth GmbH, Karlsruhe, Germany) at 37°C. Following this, individual colonies were selected and inoculated into LB broth, which was then incubated at 37°C overnight.

**Table 1:** Origin and serotype of *Salmonella* strains.

**Serotype/ designation**	**Isolation year**	**Strain *N***	**Origin**
*Salmonella* Enteritidis/a	2019	SA00115	Frozen raw chicken meat
*Salmonella* Enteritidis/b	2020	SA02231	Poultry meat
*Salmonella* Typhimurium/b	2020	SA02878	Frozen poultry meat
*Salmonella* Infantis/b	2020	SA02511	Broilers; skin with fat
*Salmonella* Paratyphi B/b	2020	SA01326	Frozen chicken lower leg
*Salmonella* Indiana/b	2019	SA02184	Frozen raw chicken meat

### 2.2 Origin and enumeration of bacteriophages

The six phages of the cocktail “Applied Phage Meat S2” were isolated from environmental sources (duck pond in Hamm, Germany and sewage treatment plant in Hamm, Germany) by the PTC Phage Technology Center (Bönen, Germany). The phages of this cocktail have been deposited in the German Collection of Microorganisms and Cell Cultures (DSMZ) and are listed in Table 2. “Applied Phage Meat S2” is a commercially available product (FinkTec GmbH, Hamm, Germany), holding an US FDA “Generally Recognized as Safe (GRAS)” status for its application to combat *Salmonella* during both meat and vegetable processing (FDA, 2023a,b). The cocktail covered over 400 *Salmonella* isolates belonging to 41 different serotypes (FDA, 2023a).

**Table 2:** Phages in the cocktail.

**Phage**	**DSM number**
vB_SalS_OBO18	DSM 33041
vB_SalM_RMS3b	DSM 33043
vB_SalS_RMP9	DSM 26157
vB_SalM_MP82	DSM 26173
vB_SalM_TAT2F	DSM 33044
vB_SalM_DIN2	DSM 33045

The phage cocktail was enumerated using a double agar overlay plaque assay (Kropinski et al., 2009). For each strain, the phage cocktail titer was determined (Table 1). Serial dilutions of phages were prepared in sodium magnesium buffer (SM, 50 mM Tris-HCl, 100 mM sodium chloride, 8 mM magnesium sulfate, pH 7.5). Overnight culture and each serial dilution of lysates (each 0.1 ml) were mixed and incubated for 5 min. Next, 5 ml of LB soft agar (0.6%) were added to the mixture and poured onto a LB plate. The LB plates were incubated at 37°C overnight.

### 2.3 Application of the phage cocktail

To apply phages on chicken skin, 10 ml spray bottles (NeoLab Migge GmbH, Heidelberg, Germany) were used. The spray volume was initially determined at least 100 times to ensure the precise quantity of phages used for application. The average value of this measurement was 129 μl (±0.01 μl). We also determined the optimal distance between the spray bottle and skin surface. Sterile water mixed with bromophenol blue was used for this purpose and sprayed onto a filter paper. The ideal spraying distance was 18 cm, resulting in a circular coverage of ~5 cm diameter.

### 2.4 Reduction of *S*. Enteritidis on chicken skin

Fresh chicken was purchased on the day of the experiment from a local supermarket. The experiment was conducted with major modifications based on the description provided by Duc et al. (2020). The breast skin was carefully removed and cut into 4 × 4 cm^2^ pieces. First two different strains of *S*. Enteritidis (strain a and strain b, Table 1.) were used. For individual strains, a 1:100 dilution of the overnight culture was prepared. The cultures were incubated at 37°C with shaking at 200 rpm. After reaching an optical density at 588 nm (OD_588_) of 0.2 (corresponding to ~5 × 10^7^ CFU/ml) each culture was diluted 10-fold and 0.1 ml of the dilution (10^−1^) was dropped onto the skin and spread with a Drigalski spatula to obtain ~5 × 10^4^ CFU/cm^2^. The artificially contaminated skin was left to dry completely under a clean bench, which took 20–90 min depending on the skin structure. Once the bacterial culture completely dried on the skin, the phage cocktail was sprayed onto the sample. A 10 mL spray bottle was fixed on a stand at a distance of 18 cm, and the skin was sprayed two to three times. The phage cocktail titer was determined for each strain and the concentration of *S*. Enteritidis a and *S*. Enteritidis b on the skin reached a final concentration of 3 × 10^6^ PFU/cm^2^ [multiplicity of infection (MOI) 50] and 2 × 10^7^ PFU/cm^2^ (MOI 500), respectively. Controls were sprayed in the same way with PBS buffer (phosphate-buffered saline). After spraying the phage cocktail, the samples were incubated at room temperature (RT) for 2, 4, 6, and 24 h.

### 2.5 Phage cocktail stability on chicken skin

Following the experiment with the two *S*. Enteritidis strains, the stability of the phage cocktail was determined. For this, after each time point (2, 4, 6, 24 h), a 1 ml aliquot (after homogenization) of the treated sample of each strain was centrifuged at 11,000 × *g*, and the supernatant was filtered through a 0.22 μm pore-size filter (VWR International GmbH, Darmstadt, Germany). The filtered lysate was titrated using the double-agar overlay plaque assay. The resulting phage titer was then compared with the initial titer.

### 2.6 Evaluation of the phage cocktail for the reduction of *Salmonella* on chicken skin

The experiment with a mixture of strains was conducted in the same way as described above for *S*. Enteritidis. However, here five different *Salmonella* serotypes were used: *S*. Enteritidis*, S*. Typhimurium, *S*. Infantis, *S*. Paratyphi B and *S*. Indiana (all strains except for *S*. Enteritidis a—see Table 1). LB broth was inoculated with 20 μl of an overnight culture (OD_588_ approx. 1.6) of each strain. The culture containing several strains was allowed to grow until it reached an OD_588_ of 0.2. The final concentrations of the bacteria and of the phage cocktail on skin was 5 × 10^4^ CFU/cm^2^ and 3 × 10^6^ PFU/cm^2^ (MOI 50), respectively. The experiment was performed in triplicate. After spraying the phage cocktail or PBS, the treated samples and controls were incubated at room temperature for 0.5, 1, 2, and 4 h.

### 2.7 Reduction experiments on stainless steel

Stainless steel plates 4 × 4 cm^2^ (N 1.4301) were produced by the Ottim Metall GmbH, Berlin, Germany. To mimic a contamination as applied in the chicken skin experiments, a single strain (*S*. Enteritidis b) was prepared. In the first experiment we simulated a new surface contamination prior to drying. In this case, the phage cocktail was sprayed onto liquid *S*. Enteritidis before being dried on stainless-steel. An amount of 0.1 ml *S*. Enteritidis b (5 × 10^5^ CFU/cm^2^) was distributed on the stainless-steel surface in small drops and was directly treated with two–three sprays of the phage cocktail (2 × 10^7^ PFU/cm^2^). In another experiment, we used a single strain (*S*. Enteritidis b was used as the positive control) and a mixture of the five serotypes. The experiment was carried out as described above. However, after contamination of the stainless-steel, *Salmonella* cells were allowed to dry completely under a clean bench. Upon drying (~20 min), the phage cocktail was sprayed onto the surface to achieve 2 × 10^7^ PFU/cm^2^ (MOI 50). This experiment was carried out in triplicate and the control samples were sprayed with PBS.

### 2.8 Determination of bacterial numbers

After the treatment, the samples of chicken skin or stainless steel and their respective controls were placed in a cold storage room (4°C). In the cold room, the samples were placed in 15 × 80 cm stomacher bags containing 20 ml of PBS. The samples were gently massaged by hand and homogenized for 1 min at level 3 (MiniMix, INTERSCIENCE, Wiesbaden, Germany). Thereafter, the samples were taken to the laboratory in an ice-bath box and diluted to a 10^−3^ dilution level. All dilutions and the undiluted sample were plated on XLD plates (Xylose Lysine Deoxycholate agar, Oxoid Deutschland GmbH, Wesel, Germany).

### 2.9 Statistical analysis

The effect of exposure to the phage cocktail was tested using paired sample *t*-test as provided by SPSS (IBM SPSS statistics, Version 26). Pairs were defined by time points per trial. Comparisons were done per trial including all four–five time points. When three treated samples were compared to one control, three comparisons were carried out.

## 3 Results

### 3.1 Reduction of two single *S*. Enteritidis strains on chicken skin

**Figure 1:**
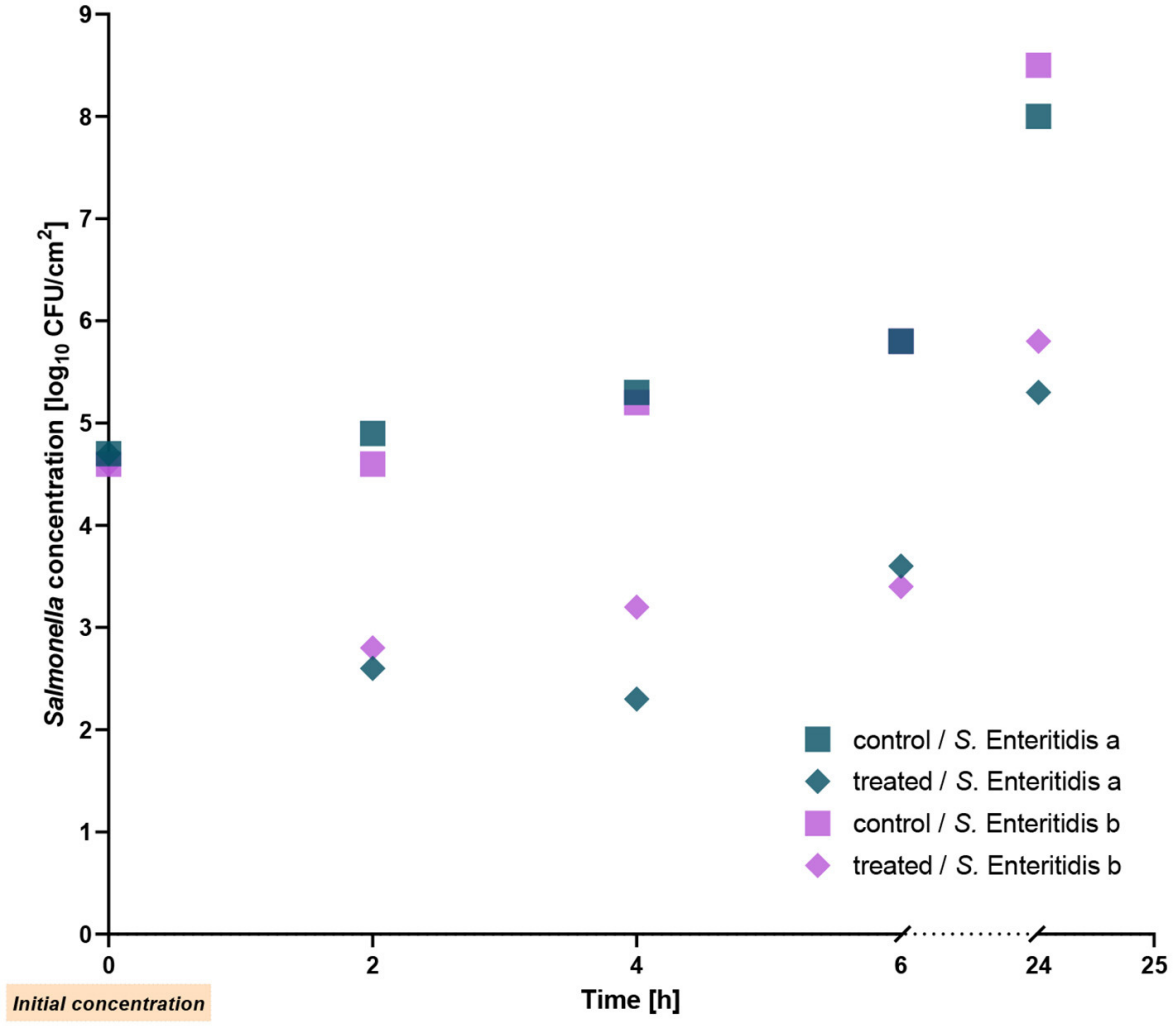
Reduction of two *Salmonella* Enteritidis strains on chicken skin by the phage cocktail at room temperature.

In a previous study performed under *in vitro* conditions, reduction of *Salmonella* strains by the phage cocktail investigated here has been demonstrated (Gvaladze et al., 2023). To examine the reduction on chicken skin, we initially tested two *S*. Enteritidis strains revealing the highest susceptibility to the phage cocktail. Reduction rates for both strains were similar, ~2 log_10_ units 2 h after addition of the phage cocktail at RT. Reduction of bacterial counts was also determined 4 and 6 h post treatment. The strongest decrease was observed after 4 h, which showed a 3 log_10_ unit reduction. Even after 24 h of treatment with the phage cocktail, a reduction of ~2.7 log_10_ units was observed for both strains (Figure 1). The results reached statistical significance with a *p-*value of ≤ 0.002. We also determined the bacterial count after-phage cocktail treatment relative to the initial count. The *Salmonella* count was 1.2 log_10_ units lower than the initial level after 6 h of treatment, whereas after 24 h similar numbers of bacteria were determined.

**Figure 2:**
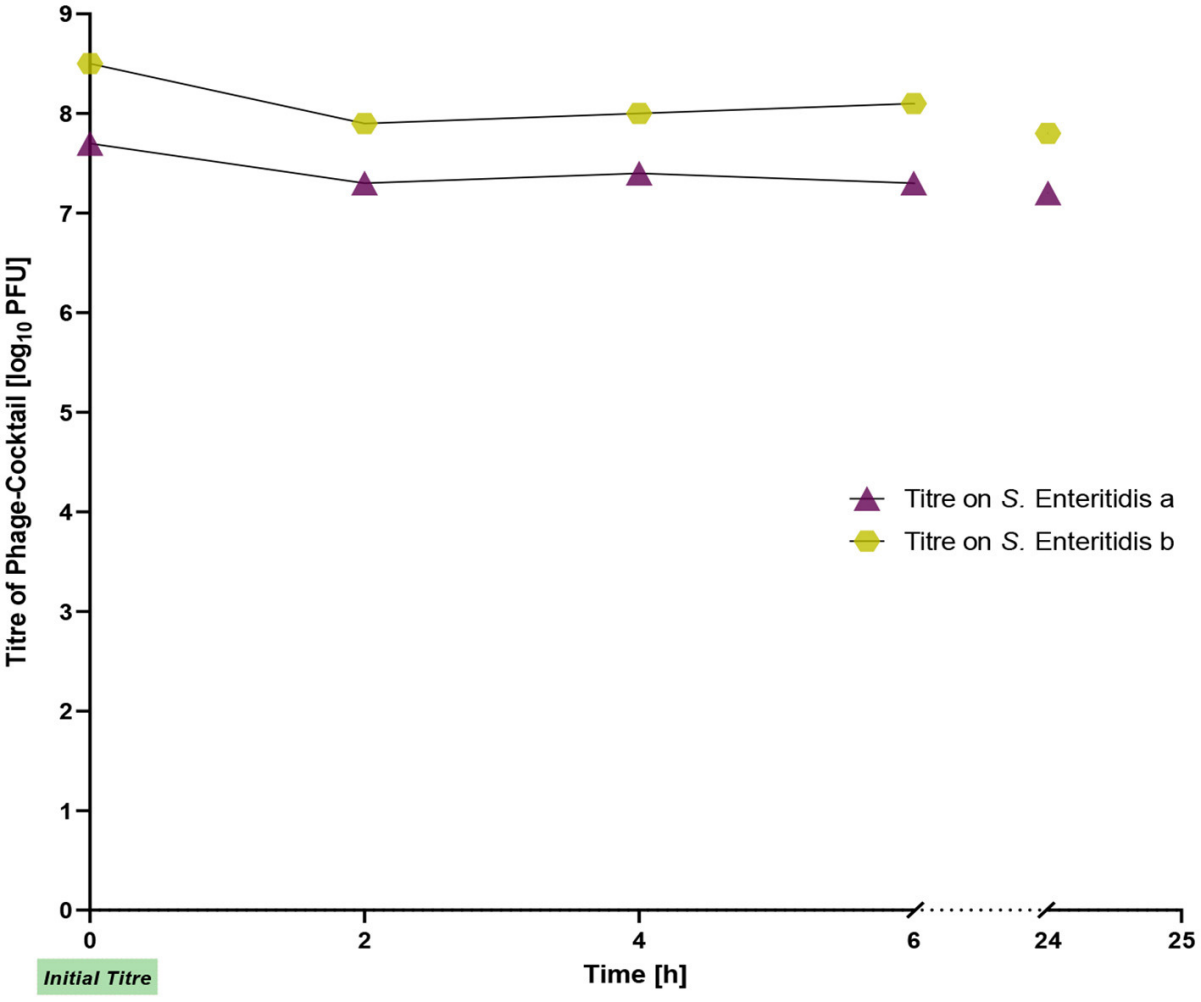
Titer of the phage cocktail on chicken skin during incubation for 24 h.

We also examined the stability of the phages on chicken skin. The initial titers of the phage cocktail for *S*. Enteritidis a and *S*. Enteritidis b were 7 × 10^7^ PFU/ml and 5 × 10^8^ PFU/ml, respectively. Throughout the entire experiment, the titers remained stable and showed only a slight decrease of ~0.5 log_10_ units (Figure 2).

### 3.2 Reduction of multi strain mixture containing five *Salmonella* serotypes on chicken skin

**Figure 3:**
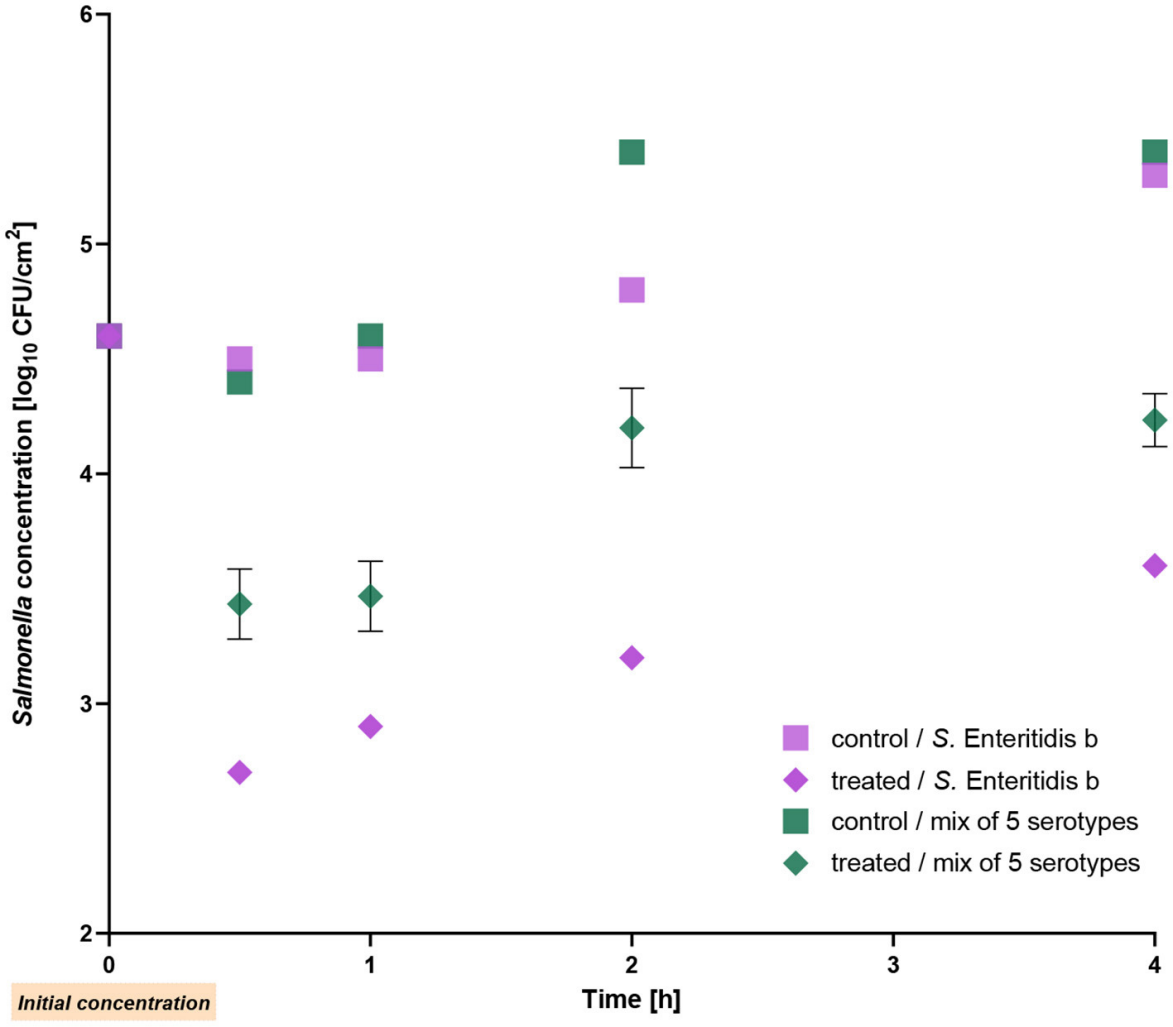
Reduction of a mixture of five *Salmonella* serotypes on chicken skin by the phage cocktail at room temperature. *Salmonella* Enteritidis strain b was used as a positive control. The error bars in the figure represent the mean and standard deviation (SD) for three phage-treated samples.

In this experiment, the reduction of a mixture of five different *Salmonella* serotypes was studied. *S*. Enteritidis strain b was used as the positive control. The mixture showed a reduction of 1 log_10_ unit after 30 min of phage application, and the reduction remained stable throughout the entire experiment, resulting in a decrease of 1.2 log_10_ units (*p* ≤ 0.002) after 4 h incubation at RT. On the other hand, the single Enteritidis strain b was reduced by 1.5–1.8 log_10_ units during the 4 h (*p* < 0.001; Figure 3). Compared to the initial count, the bacterial counts of *S*. Enteritidis b and of the mixture were also lower (up to 1 log_10_ unit) after treatment for 4-h.

### 3.3 Reduction experiments on stainless steel

**Figure 4:**
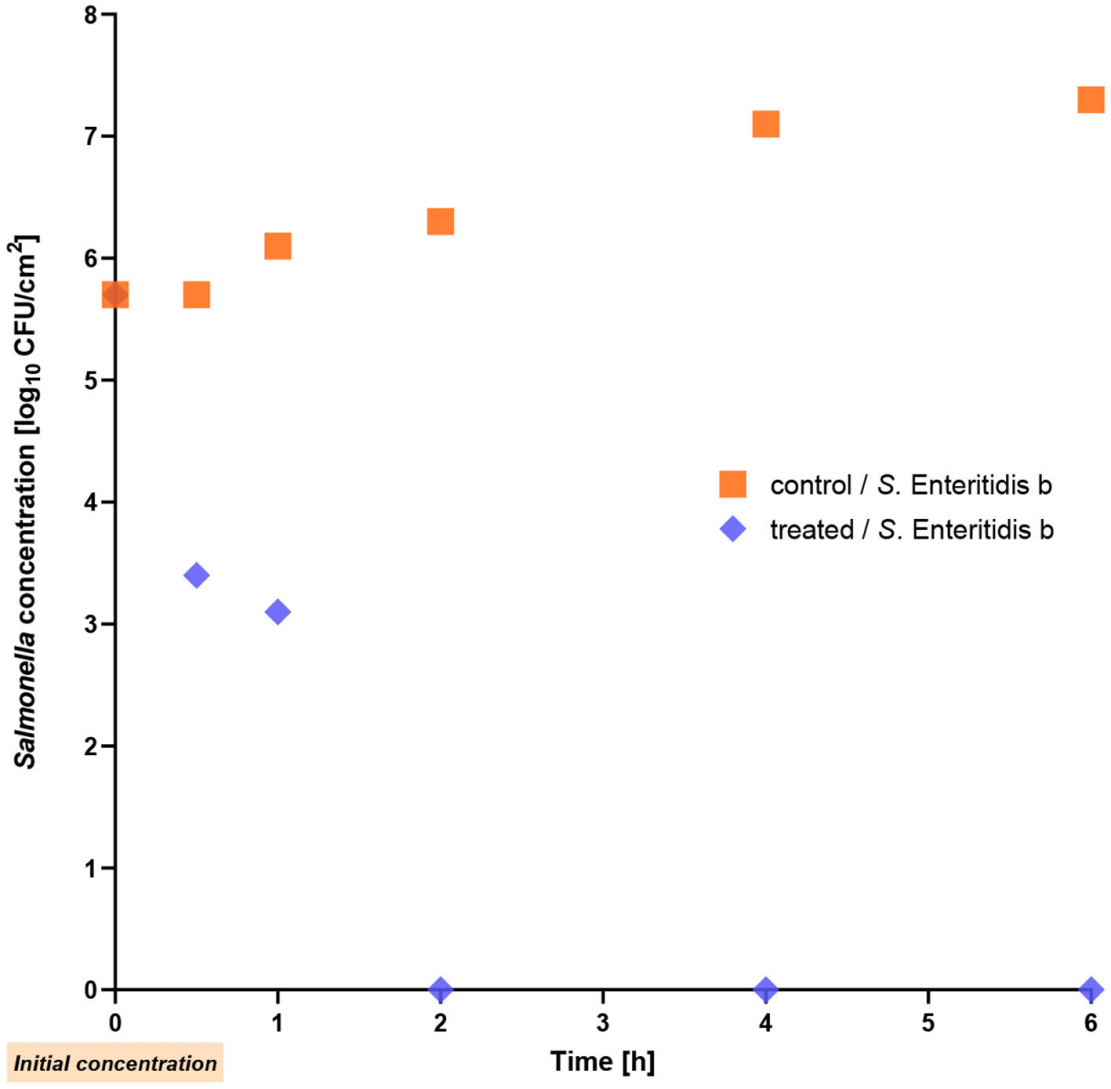
Reduction of *Salmonella* Enteritidis strain b on stainless-steel at room temperature. The phage cocktail was applied to the still wet contamination.

We now wanted to learn whether a significant reduction of *Salmonella* is also achievable on a stainless steel surface. The first thing we did was to simulate a new surface contamination prior to drying. In this case, the phage cocktail was used to infect liquid *S*. Enteritidis. The reduction on wet stainless steel was much higher than lysis after drying the bacteria (see below). Within the first hour of treatment, the single *S*_._ Enteritidis strain b exhibited a remarkable decrease of 3 log_10_ units (*p* = 0.012) and after 2 h, the bacterial count reached a level below the detection limit (Figure 4).

**Figure 5:**
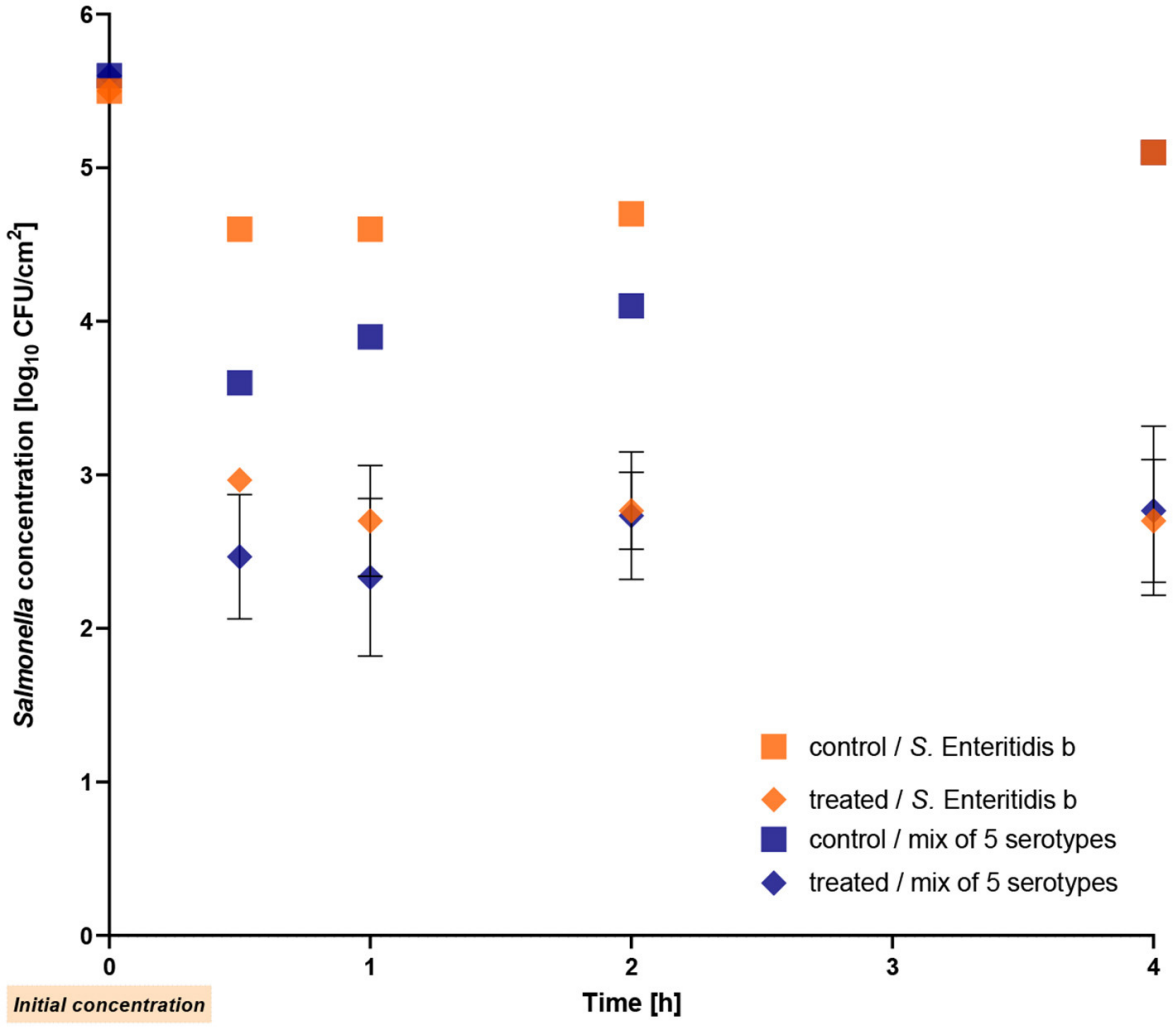
Reduction of the mixture of five *Salmonella* serotypes after drying on stainless-steel by the phage cocktail at room temperature. The *Salmonella* Enteritidis strain b was used as a positive control. The error bars in the figure represent the mean and standard deviation (SD) for three phage-treated samples.

In the second experiment, the bacteria were allowed to dry completely on the stainless steel surface before adding the phages. The experiment was performed with *S*. Enteritidis strain b (positive control) and the mixture containing the five different serotypes. Figure 5 illustrates the obtained results indicating a similar reduction of both the single *Salmonella* strain and the mixture, with reductions of up to 2.4 log_10_ units (*p* ≤ 0.009) observed after 4 h of treatment. Whereas the mixture exhibited a reduction of 1.2 log_10_ units within the first 30 min, the single strain showed a reduction of 1.7 log_10_ units.

## 4 Discussion

In this study, we analyzed the effectiveness of a phage cocktail for the reduction of relevant *Salmonella* serotypes on both chicken skin and stainless-steel surfaces. Our findings clearly demonstrate that this phage cocktail could be a promising biocontrol tool, which could be employed for post-harvest applications during poultry production. Initially, the ability of the phage cocktail to reduce the predominant serotype *S*. Enteritidis was determined. Thereafter, its performance against a mixture of five important *Salmonella* serotypes (*S*. Enteritidis, *S*. Typhimurium, *S*. Infantis, *S*. Paratyphi B and *S*. Indiana) was examined. Chicken can contain multiple *Salmonella* serotypes simultaneously (Betancor et al., 2010). The coexistence of multiple *Salmonella* serotypes in chicken and poultry poses a significant challenge to food safety. A phage cocktail can effectively target multiple serotypes; therefore, it is common to use two or more phages for applications (Sevilla-Navarro et al., 2018). The cocktail comprising six-phages showed a significant reduction of the single *Salmonella* strain as well as of the mixture on both matrices. Slaughterhouses being potential sources of *Salmonella* cross-contamination of chicken meat play a pivotal role in controlling the spread of this pathogen throughout the food chain (Heyndrickx et al., 2007; Zeng et al., 2021). While in numerous studies the application of *Salmonella* phages has been explored on food samples spiked with a single serotype, there are few reports on the reduction of mixtures of different *Salmonella* serotypes. In addition, there is only a limited number of studies employing commercial phage cocktails against mixtures of bacteria. Yeh et al. (2017) applied two commercial phage cocktails at a concentration of up to 10^8^ PFU/ml during the tumbling process to reduce a mixture of four *Salmonella* serotypes (*S*. Enteritidis, *S*. Infantis, *S*. Heidelberg, and *S*. Newport) in various ground meat types, including chicken, at 4°C. This application resulted in a reduction of ~1 log_10_ unit. Sukumaran et al. (2015) and Yeh et al. (2018) also compared the application of individual or combined commercial phage cocktails with chemical and/or physical methods to reduce mixed *Salmonella* serotypes. In both studies reductions of up to 1 log_10_ unit were achieved. In another study, Sukumaran et al. (2016) compared dip and surface applications of the commercial phage cocktail SalmoFresh™ on chicken breast using a three-serotype cocktail (*S*. Typhimurium, *S*. Enteritidis, and *S*. Heidelberg). They again achieved reductions of up to 1 log_10_ unit at 4°C and RT. According to this result, dipping of food in a phage solution does not achieve better reductions than spraying. Grant et al. (2017) used the Salmonelex™ phage cocktail to reduce a *Salmonella* contamination on two different bacterial mixtures: the first mixture contained *S*. Newport, *S*. Typhimurium, and *S*. Thomson while the second mixture comprised *S*. Enteritidis, *S*. Heidelberg, and *S*. Typhimurium. They achieved maximum reductions of 0.9 log_10_ units and 0.7 log_10_ units at 4°C after 8 h, respectively. The bacterial and phage doses used in these experiments were similar to those used in our study, with 10^4^ CFU and 10^7^ PFU per cm^2^ of chicken meat. Whereas almost all of these bacterial mixtures included *S*. Enteritidis or *S*. Typhimurium, Yeh et al.'s mixture was the only one that contained the relevant serotype *S*. Infantis. We went a step further by testing a combination of five different serotypes. As far as we are aware, no comparable results have been published as yet.

The use of phages on stainless steel remains an area of limited research. Stainless steel, a commonly used material in food processing equipment (Veluz et al., 2012), can act as a carrier of bacteria, thereby facilitating transmission of pathogens and cross-contamination (Arnold and Silvers, 2000). To the best of our knowledge, only a few studies have explored the reduction of *Salmonella* using phages on stainless-steel surfaces (Woolston et al., 2013; Gong and Jiang, 2017; Ge et al., 2022; Korzeniowski et al., 2022). Korzeniowski et al. (2022) investigated the impact of individual phages and a cocktail at various concentrations on *S*. Enteritidis biofilms, resulting in reductions of 60%−97% at 37°C. Ge et al. (2022) contaminated a metal surface with *S*. Enteritidis and applied a single phage, achieving a 1 log_10_ unit reduction. Woolston et al. (2013) employed a commercially available phage cocktail, similar to our approach. The cocktail, SalmoLyse™, reduced a surface contamination with Paratyphi B by 2.1–4.1 log_10_ units. Gong and Jiang (2017) studied a six-phage cocktail against *S*. Typhimurium and a mix of 10 *S*. Typhimurium isolates, with reductions of 3 log_10_ and 2 log_10_ units, respectively at 23°C. The last study is consistent with our results in the manner that it shows a greater reduction with a single strain. Though, even when mixed strains were used, significant reductions were observed.

In conclusion, our study demonstrated the effectiveness of a phage cocktail on both single *S*. Enteritidis strains and a mixture of five different *Salmonella* serotypes. Moreover, reductions persisted over an extended period. In a previous study, we assessed the impact of the phage cocktail at low temperatures ranging from 15 to 6°C, using individual and mixed *Salmonella* cultures. The significant reduction of a mixture of the five different *Salmonella* serotypes at 6°C suggests that the phage cocktail remains active within the cold chain. Resistance against the six phages was not observed, we identified at least one phage to which the bacteria exhibited sensitivity (Gvaladze et al., 2023). Thus, it is likely that some bacteria survived the infection not because of resistance, but rather because of the fact that they entered the late logarithmic or even stationary growth phase, in which propagation of phages is limited. It is of course also conceivable that they simply survived, because they did not encounter an infectious particle. The promising results of this study corroborate the potential of these phages for applications in post-harvest settings such as slaughterhouses under real conditions.

## Data availability statement

The original contributions presented in the study are included in the article/supplementary material, further inquiries can be directed to the corresponding author.

## Author contributions

TG: Data curation, Formal analysis, Investigation, Methodology, Project administration, Resources, Validation, Visualization, Writing – original draft, Writing – review & editing. HL: Conceptualization, Funding acquisition, Project administration, Resources, Validation, Writing – review & editing. SH: Conceptualization, Funding acquisition, Methodology, Project administration, Supervision, Validation, Writing – original draft, Writing – review & editing.
